# Intoxication due to Δ9-tetrahydrocannabinol is characterized by disrupted prefrontal cortex activity

**DOI:** 10.1038/s41386-024-01876-5

**Published:** 2024-05-07

**Authors:** Keerthana Deepti Karunakaran, Michael Pascale, Nisan Ozana, Kevin Potter, Gladys N. Pachas, A. Eden Evins, Jodi M. Gilman

**Affiliations:** 1 Massachusetts General Hospital (MGH) Department of Psychiatry, Boston, MA USA; 2 Harvard Medical School, Boston, MA USA; 3 MGH/HST Athinoula A. Martinos Center for Biomedical Imaging, Department of Radiology, Massachusetts General Hospital, Harvard Medical School, Charlestown, MA USA; 4 Faculty of Engineering and The Gonda Multidisciplinary Brain Research Center, Bar Ilan University, Ramat-Gan, 52900 Israel

**Keywords:** Optical spectroscopy, Biomarkers

## Abstract

Neural states of impairment from intoxicating substances, including cannabis, are poorly understood. Cannabinoid 1 receptors, the main target of Δ9-tetrahydrocannabinol (THC), the primary intoxicating cannabinoid in cannabis, are densely localized within prefrontal cortex; therefore, prefrontal brain regions are key locations to examine brain changes that characterize acute intoxication. We conducted a double-blind, randomized, cross-over study in adults, aged 18–55 years, who use cannabis regularly, to determine the effects of acute intoxication on prefrontal cortex resting-state measures, assessed with portable functional near-infrared spectroscopy. Participants received oral THC (10–80 mg, individually dosed to overcome tolerance and achieve acute intoxication) and identical placebo, randomized for order; 185 adults were randomized and 128 completed both study days and had usable data. THC was associated with expected increases in subjective intoxication ratings (*ES* = 35.30, *p* < 0.001) and heart rate (*ES* = 11.15, *p* = 0.001). THC was associated with decreased correlations and anticorrelations in static resting-state functional connectivity within the prefrontal cortex relative to placebo, with weakest correlations and anticorrelations among those who reported greater severity of intoxication (RSFC between medial PFC-ventromedial PFC and DEQ scores, *r* = 0.32, *p* < 0.001; RSFC between bilateral mPFC and DEQ scores, r = –0.28, *p* = 0.001). Relative to placebo, THC was associated with increased variability (or reduced stability) in dynamic resting-state functional connectivity of the prefrontal cortex at *p* = 0.001, consistent across a range of window sizes. Finally, using frequency power spectrum analyses, we observed that relative to placebo, THC was associated with widespread reduced spectral power within the prefrontal cortex across the 0.073–0.1 Hz frequency range at *p* < 0.039. These neural features suggest a disruptive influence of THC on the neural dynamics of the prefrontal cortex and may underlie cognitive impairing effects of THC that are detectable with portable imaging. This study is registered in Clinicaltrials.gov (NCT03655717).

## Introduction

Neural states of impairment from intoxicating substances, including cannabis, are poorly understood. Cannabinoid 1 (CB1) receptors are densely localized within the frontolimbic circuit, which is the main target of Δ9-tetrahydrocannabinol (THC), the primary intoxicating cannabinoid in cannabis [1]. Placebo-controlled clinical studies consistently highlight a range of cognitive and psychomotor deficits associated with acute cannabis intoxication, including deficits in attention [2], psychomotor function [3], impulse control [4], decision-making [5], and reduced ability to learn and recall new information, particularly in domains of short-term episodic and working memory (see [6] for review). Performance in each of these domains is largely mediated by the prefrontal cortex (PFC), which underlies higher-order cognitive processes (e.g. planning, organizing, regulating behavior), inhibitory control, emotional regulation, and regulating and allocating attention to relevant information while filtering out irrelevant distractions [7]. In addition, the PFC, particularly dorsolateral PFC, plays a critical role in working memory processes, e.g. the temporary storage and manipulation of information needed for cognitive tasks [8]. Thus, PFC regions are key locations to examine brain changes that characterize impaired clinical states associated with acute intoxication.

There is also a growing public health need for an objective, reliable, unbiased method to detect impairment due to acute THC intoxication [9], which can have profoundly impairing effects, especially in individuals who use cannabis infrequently [6, 10, 11]. Determination of THC impairment is not achievable with any THC or THC metabolite concentration in blood or body fluid primarily due to inter-individual pharmacodynamic variation including tolerance to the impairing effects of THC and also due to the time course for clearance of THC and its metabolites [12]. Some individuals, even with oral THC doses up to 80 mg do not report subjective or exhibit clinical signs of impairment [13] due to the development of tolerance from daily use [14]. Thus, reliable objective indictors of impairment, rather than measures of exposure, are needed to determine impairement due to THC intoxication. Functional near-infrared spectroscopy (fNIRS), a noninvasive and inexpensive method for assessing cerebral hemodynamics (oxy-hemoglobin (HbO), and deoxy-hemoglobin concentration changes) as a measure of neuronal activity, can be used to query the neurovascular response to THC. fNIRS experiments are increasingly performed outside the laboratory in everyday life situations [15–17]. This method is portable [18] and requires minimal set-up time [19], making it ideal for use in real-world settings [20].

We previously reported that task-induced changes in cerebral hemoglobin concentration were significantly altered by THC intoxication during an n-back working memory task [21, 22], and that this change could be used to predict acute impairment in individual participants [23], suggesting that a neural activity signature of THC intoxication was detectable with portable fNIRS brain imaging. Studies, however, have not used fNIRS to investigate the neurobiolgical effects of THC on the resting-state brain architecture, which are measures of the intrinsic, spontaneous activity and functional connections between different brain regions while an individual is at rest or not engaged in a specific task [24]. This is particularly needed, as studies using fMRI report that THC-induced changes in behavior are likely related to the effects of THC on RSFC. One study reported THC causing widespread reductions in functional connectivity in the left frontal (dorsolateral prefrontal cortex (DLPFC), ventrolateral prefrontal cortex (VLPFC)) and temporal (anterior superior temporal gyrus, posterior inferior temporal gyrus) regions [25]. Reduced functional connectivity within the mesocorticolimbic and salience network were associated with decrements in cognitive function and increments in psychotomimetic symptoms [26]. Decreased functional connectivity was particularly prominent in the (supracallosal) anterior cingulate and medial prefrontal cortex (MPFC) [4]. These are consistent with task-evoked fMRI studies showing that THC causes increased dopamine release via activation of presynaptic CB1 receptors in the ventral tegmental area, via GABAergic terminals [27], resulting in reduced functional connectivity in the mesocorticolimbic circuit [28], which can in turn negatively impact cognition. For example, THC-induced changes in response latency during attentional tasks are related to modulations in PFC and striatal activation that result in decreased mesocorticolimbic functional connectivity [29], and THC-induced deficits in psycho-motor tasks are associated with altered activity within the mesocorticolimbic circuit and salience network [30]. A similar study reported that impaired task performance after THC administration was associated with reduced deactivation in the default mode network [31].

While existing research offers a detailed understanding of the impact of THC on brain state through the use of fMRI studies, the practicality of fMRI outside laboratory environments is limited. Further, evaluation of resting-state functional connectivity (RSFC) and other intrinsic measures using fNIRS could prove valuable in settings where completing a task may be difficult, like at the roadside. Here we investigate the effect of acute THC on the resting-state characteristics of PFC function using fNIRS. To do so, we conducted a double-blind, placebo-controlled, crossover study, randomized for order, in otherwise healthy adults who reported at least weekly cannabis use. First, we investigated the effect of oral THC on static connectivity within the PFC to delineate the regions that are most affected by THC-induced changes and to replicate the existing literature using fNIRS. To extend upon existing literature, we then sought to characterize variability in dynamic RSFC (dRSFC) within the PFC. dRSFC is a relatively new method of assessing temporally correlated activation states of discrete brain regions over time [32], assessing variability in the strength and spatial organization of functional connectivity across brain regions, and can be used to elucidate the temporal dynamics of connectivity changes and their impact on neural network stability. We previously reported, in a small sample (*n* = 41), that machine learning metrics could be used to predict intoxication from dRSFC measures [33]. Here, we expand this finding in a larger sample size and with the intent to describe neurobiology underlying these observations. Finally, to further understand the effect of THC on intrinsic activity, we examined THC-induced changes in spectral power, a measure of the distribution of signal power across different frequencies within a given signal (e.g., changes in Hb oxygenation over time). Here we focused on potential alterations in signal power across different frequencies within a given signal, with the aim of assessing whether changes in average spectral power across specific frequency bands could be indicative of alterations in functional connectivity patterns. Together, an understanding of the effects of THC on static RSFC, stability of dRSFC, and power spectral density, could provide a more comprehensive understanding of the impact of THC intoxication on prefrontal cortical function, as each of these alterations could potentially underlie the cognitive effects of acute intoxication. Detection of these effects using fNIRS could further the science on an objective measure of intoxication from THC.

## Materials and methods

Study procedures were approved by the Mass General Brigham Human Subjects Committee, and all participants provided written informed consent before initiation of these procedures. The clinical trial is registered in Clinicaltrials.gov and is available online (NCT03655717).

### Participants

Adults, aged 18–55 years, who reported cannabis use weekly or more in the 90 days before enrollment, were recruited through community advertising between January 2017 and March 2020 at Massachusetts General Hospital (MGH). Exclusion criteria included a negative urine THC (THCCOOH) screen (20 ng/mL cutoff; Medimpex United Inc., Bensalem, PA), serious unstable medical illness, lifetime history of schizophrenia or bipolar disorder, current regular use of benzodiazepines or barbiturates, and known allergy to dronabinol or its constituents. Participants were compensated for completing each study visit. Urine was collected at the screening visit for quantitative using creatinine-adjusted 11-nor-9-carboxy-Δ9-tetrahydrocannabinol metabolite concentration (Dominion Diagnostics, Kingstown, RI, USA) [34] to rule out participants without recent cannabis use.

### Study design

This was a double-blind, placebo-controlled, cross-over design clinical trial in which participants were randomized for order for single dose synthetic THC and identical placebo on separate study visits at least 7 days apart (mean = 10.1 days, SD = 10.9 days). The randomization was performed by the MGH Clinical Trial Pharmacy. Participants were instructed to abstain from cannabis for 24 h prior to experimental sessions. Participants underwent a qualitative urine drug screen and clinical evaluation for signs of acute intoxication before each study visit and were rescheduled if they screened positive for any drug except cannabis or showed signs of acute cannabis intoxication. Participants received a standard 400-calorie breakfast before dosing. The participants, investigators and study staff were blinded to the assigned intervention.

### Study drug and administration

THC was administered as dronabinol capsules (Marinol), an FDA-approved synthetic THC. Study physicians asked participants about regular patterns of use and determined the approximate usual dose of THC each participant regularly used that produced intoxication without adverse events such as anxiety, panic, paranoia, and hemodynamic change [21]. Doses of 5 mg to 80 mg were administered.

### Physiology and Perceptions of Intoxication

Subjective ratings of drug effects (Drug Effects Questionnaire; DEQ) [35] and heart rate (HR) were collected once before administration of study drug and at intervals of approximately 20 min after drug administration. The DEQ consists of five questions, in which participants rate perceived effects of the study drug from zero (no effect) to 100 (maximum effect). The DEQ item “How high are you high right now?” was used to assess self-reported feelings of intoxication.

### Resting-state fNIRS

Participants underwent a 6 min, resting-state fNIRS scan before and approximately 100 and 200 min after study drug administration on both study days. The timing of the second scan (Post-dose Scan 1) was planned to coincide with the estimated median maximum THC concentration in blood, and thus, we intended for these scans to be used in the current analyses. Because the time course of intoxication was variable, likely due to oral dosing, some participants were not intoxicated at Scan 1 but became intoxicated later in the study. Therefore, we included the scan in which the participants reported the highest DEQ rating of ‘intoxicated’ in the analyses. Resting-state scans were obtained in a dark room.

During each scan session, a continuous-wave fNIRS device (NIRSport 8-8, NIRx Medical Technologies LLC, Glen Head, NY, USA) was placed over the forehead. A configuration of eight sources and seven detectors was used, providing 20 channels over the PFC region. The NIRS device was configured to acquire dual wavelengths (760 and 850 nm) at 7.81 Hz. The cap was placed over each participant’s vertex, at a point equidistant from the nasion and inion and equidistant from the left and right preauricular points. The mid-column of the probe was placed over Fpz, and the lowest probes were along the F5-Fp1-Fpz-Fp2-F6 line of the International 10–20 System [36]. Source-detector separations were between 2.5 cm and 3 cm with channels defined at the midpoints of these pairs.

### fNIRS data analysis

#### Preprocessing

The fNIRS data were preprocessed using a standard pipeline in the open-source toolbox Homer2 [37] on MATLAB platform (Mathworks, Natick, MA). The first step of preprocessing involved the conversion of raw light intensity timeseries from the NIRS device to optical density measures. Savitzky-Golay smoothing (available in Homer2) was applied to correct for head motion artifacts, and a Butterworth band-pass filter of 0.01–0.5 Hz was applied to reduce baseline drift, the impact of cardiac activity, low frequency oscillations, and high frequency noise [38]. The motion-corrected and filtered optical density values were converted to relative changes in oxygenated (ΔHbO) and deoxygenated (ΔHbR) hemoglobin concentrations based on the modified Beer-Lambert law [39]. Finally, the first principal component from all channels was removed using a linear regression to reduce influence of non-neuronal physiologic factors on the data [40], and the primary and secondary outcome measures were calculated. Changes in oxy-hemoglobin concentration pre- vs. post-drug states were defined as primary outcome measures, and have been reported previously [23]. For the current analysis, metrics of the oxy-hemoglobin concentration change during resting-state, namely, static functional connectivity (RSFC), dynamic functional connectivity (dRSFC), and amplitude of low-frequency fluctuations (ALFF) were defined. Fluctuations in oxy-hemoglobin concentration at rest i.e., in the absence of an exogenous task, are thought to reflect cerebral blood flow changes resulting from the metabolic demands of intrinsic neuronal activity. This is based on the principle of neurovascular coupling, where an increase in neural activity leads to an increased consumption of oxygen compensated by increase in blood flow to the active area of the brain. This increased blood flow brings more oxygenated hemoglobin to the area, which is necessary to meet the higher metabolic demands of active neurons. Thus, resting-state metrics of fNIRS signal provide indirect measures of spontaneous neuronal oscillations on the basis of neurovascular coupling [41].

#### Static resting-state functional connectivity (RSFC)

A channel-to-channel pairwise connectivity of ΔHbO timeseries at the 0.01–0.1 Hz range was performed to characterize the RSFC profile within the PFC. The functional connectivity between a channel pair was defined as the pair-wise Pearson’s r correlation coefficient between the two timeseries. A 20 × 20 correlation matrix was generated for each participant. The functional connectivity measures were then normalized using a Fisher r-to-z transformation, resulting in a 20 × 20 fisher-z matrix for each participant.

#### Variability in dynamic resting-state functional connectivity

Dynamic RSFC (dRSFC) was computed using a sliding window correlation analysis [42] where pairwise correlation analysis as performed for smaller windows of 300 samples (38.4 s) that were incremented by 100 samples (6.4 s) until end of the time series. The standard deviation of the resulting timeseries (i.e., dynamic connectivity) was defined as variability in dRSFC. Effects of window size were evaluated by repeating the analysis for seven different windows (100, 200, 300, 400, 500, 600, and 700 samples) with increments of 50 samples.

#### Amplitude of intrinsic low-frequency signal

Amplitude of low frequency fluctuations (ALFF) is a measure of the average spectral power in the resting-state low-frequency oscillations [43]. It was defined as the regional intensity of ΔHbO time series at specific frequency bins, namely SLOW-5 (0.01–0.027 Hz), SLOW-4 (0.027–0.073 Hz), and SLOW-3 (0.074–0.1 Hz) [44–46], and calculated using the Eq. (1) below,

1 $${ALFF}=\frac{{\sum }_{{i}_{{low}}}^{{i}_{{up}}}\sqrt{{{{{{{\rm{|}}}}}}{FFT}(x){{{{{\rm{|}}}}}}}^{2}}}{n}$$

where, FFT(x) is the Fast Fourier Transform of ΔHbO timeseries x, i_low_ to i_up_ denote the predefined lower and upper cut-off of the frequency interval (e.g., 0.01 to 0.027 Hz for SLOW-5) and n is the number of elements in that frequency interval. The ALFF measures of all 20 channels in each of the three frequency bins were computed for each subject.

### Statistical Analysis

We first conducted a one sample t-test on the channel-wise connectivity matrices during each condition (pre-placebo, post-placebo, pre-THC and post-THC) to visualize the group-level connectivity maps within the PFC. Channel-level alterations in each resting-state measure (RSFC, variability in dRSFC and ALFF at SLOW-5, SLOW-4 and SLOW-3 bins) were then evaluated using a repeated measures ANOVA, defined with two factors, Drug (THC, Placebo) and Time (Pre-drug, Post-drug). The pre-drug versus post-drug differences following THC versus placebo was examined with Drug x Time interaction effects. Association between RSFC and severity of intoxication (DEQ intoxication rating) was evaluated using linear regression. A *p* < 0.05 statistical threshold was implemented for all analyses. Multiple comparison problem and the risk of Type-I error was mitigated using a Benjamini-Hochberg False Discovery Rate (FDR) based approach with alpha=0.05 [47]. Due to the lack of preliminary data on effect sizes of THC on fNIRS measured- PFC RSFC, no a priori power analysis was performed.

## Results

### Participant characteristics

One hundred twenty-eight participants completed two study days with sufficient fNIRS scan quality. See Supplementary Fig. 1. Participants included 62 females [48.4%]; mean [SD] age, 25.4 [6.4] years, 74.8% white, 10% black, 22% Hispanic. 72 participants (56.2%) reported daily or near-daily cannabis use, and 77 (63%) reported using multiple times per day on use days (Supplementary Table 1). One hundred and seven participants reported highest subjective intoxication (as determinded using DEQ ‘How high are you now?’ scores) nearest to Scan 1, while twenty-one participants reported highest subjective intoxication at Scan 2. Thus, for our fNIRS analyses, we chose the scan nearest in time to the peak intoxication rating.

### Acute THC effects

**Fig. 1:**
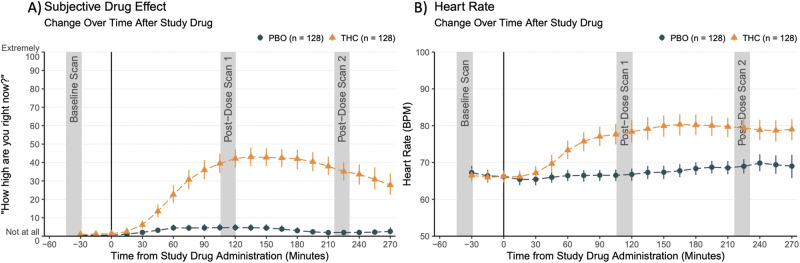
Psychological and physiological measures of THC. **A** Participants were assessed for self-reported high (0–100 scale to answer the question: “How high are you right now?”, 0 being “Not at all” and 100 being “Extremely”) at 20-minute intervals. **B** Heart rate (beats per minute) was assessed at 20-min intervals. Error bars are 95% confidence intervals for the mean. Gray bars show time windows for fNIRS imaging at baseline and post-dose scan 1 and 2. PBO = placebo.

Subjective drug effects (measured using DEQ scores of intoxication) and physiological effects (measured using heart rate) from baseline to 270 min post-drug administration are shown in Fig. 1. DEQ ratings were not different at baseline (*ES* = 0.55, *t* (116.03) = 1.51, *p* = 0.134) and significantly higher in the THC condition than the placebo condition at scans 1 and 2 (*ES* = 35.30, *t* (370.91) = 17.55, *p* = <0.001). Likewise, heart rate was not different at baseline (*ES* = –1.14, t (103.62) = –1.71, *p* = 0.090) and significantly higher in THC than placebo condition at scans 1 and 2 (*ES* = 11.15, *t* (368.88) = 13.15, *p* < 0.001).

### Static RSFC following THC

**Fig. 2:**
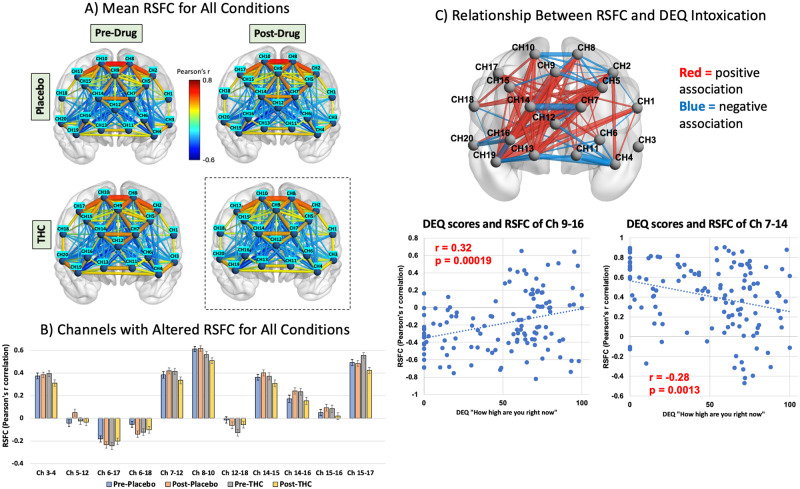
Resting-state functional connectivity after THC intoxication. **A** Group level resting-state functional connectivity during pre-drug and post-drug states for placebo and THC conditions. The color bar indicates the strength of the connectivity in Pearson’s r correlation where warm colors represent positive correlation and cold colors represent negative i.e., anti-correlation. **B** Drug x Time interaction effects at *p* < 0.05 for the repeated measures ANOVA. *p < 0.01. **C** Channel-wise brain map showing associations (i.e., Pearson’s r correlation) between post-THC RSFC and DEQ intoxication ratings that are *|r* | >0.1. Red connections represent associations that are positive, whereas blue connections represent associations that are negative. The thickness of the connections represents the magnitude of the association, with greater thickness=greater correlation. Scatter plot of the RSFC of channels 9–16 (between medial PFC and ventrolateral PFC) show positive association with DEQ ratings at FDR-*p* < 0.05 (uncorr-*p* < 0.001, *r* = 0.32). Scatter plot of the RSFC of channels 7–14 (between left and right medial PFC) shows negative association with DEQ ratings (uncorr-*p* = 0.001, *r* = –0.28).

Ten medial dorsal prefrontal (MDPFC) channels (2,5,7,8,9,10,12,14,15,17), corresponding to the anterior default mode network region, were positively correlated in all conditions (pre-placebo, post-placebo, pre-THC and post-THC), whereas longer range connections between the MDPFC channels and the ventrolateral prefrontal (VLPFC) channels (4, 6, 16, 20) were anticorrelated (Fig. 2A). This is indicative of normative activity of the DMN, as the DMN is known to be anticorrelated with the executive control network, which has nodes in dorsal PFC. There was also a notable decrease in the strength of correlations within the MDPFC channels post-THC.

Following THC administration, the RSFC in 11 channel-to-channel connections was decreased (Fig. 2B). There was a Drug x Time interaction in channel connections 3–4 (*p* = 0.043, *η*_*p*_^*2*^ = 0.031, *95% CI*_*ηp2*_: [0,0.11]), 7–12 (*p* = 0.009, *η*_*p*_^*2*^ = 0.051, *95% CI*_*ηp2*_:[0.003,0.14]), 8–10 (*p* = 0.038, *η*_*p*_^*2*^ = 0.033, *95% CI*_*ηp2*_:[0,0.112]) and 15–17 (*p* = 0.009, *η*_*p*_^*2*^ = 0.052, *95% CI*_*ηp2*_:[0.003,0.141]), where RSFC was reduced post THC but not under placebo or pre-THC conditions, suggesting that normative correlations within the anterior DMN connections at rest are suppressed in the intoxicated condition.

Impact of THC on RSFC of PFC regions was significantly associated with severity of intoxication. As shown in Fig. 2C, the greater the severity of intoxication, the greater the impact of THC on RSFC, with both correlations and anticorrelations between cortical regions being weakened in the intoxicated state. The strongest association between DEQ intoxication ratings and RSFC were observed in longer range anti-correlations within the PFC, e.g., RSFC of channels 9–16, between dorsomedial PFC and ventrolateral PFC, was positively correlated to self-reported DEQ intoxication ratings at FDR-p < 0.05 (*r* = 0.32, *p* < 0.001, 95% CI_*r2*_ = [0.02,0.22], Fig. 2C). Similarly, the strongest negative association with self-reported measures were found for interhemispheric connectivity between channels 7–14 of medial PFC such that increasing levels of intoxication was associated with reduced magnitude of correlations (*r* = –0.28, *p* = 0.001, 95% CI_*r2*_ = [0.013 0.188]). Additionally, comparing the median ΔHbO concentration between THC and placebo conditions revealed no notable changes in the median concentration of the resting-state fNIRS signal following THC (see Supplementary Fig. 2).

### Variability in dynamic RSFC following THC

**Fig. 3:**
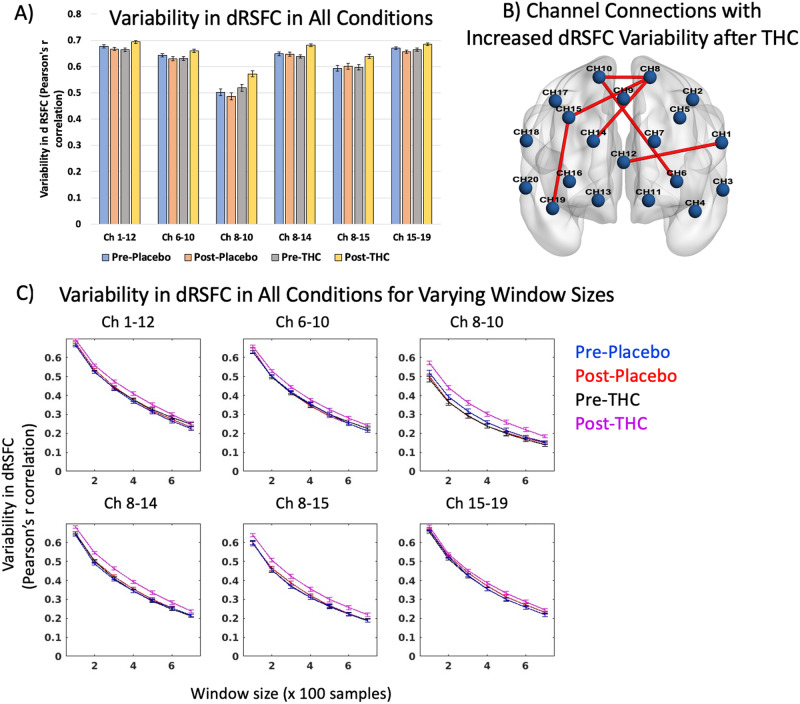
Variability in dynamic RSFC after THC intoxication. **A** Results of repeated measures ANOVA comparing variability in dRSFC between pre- and post- placebo and THC conditions at FDR-*p* < 0.05 (uncorr-*p* = 0.0015). **B** Brain map showing the location of channel pairs depicted in panel (**A**) with significant THC effects. **C** Trends in variability of dynamic RSFC for the six channel pairs in panels (**A**) and (**B**) for window sizes 100–700 samples.

**Fig. 4:**
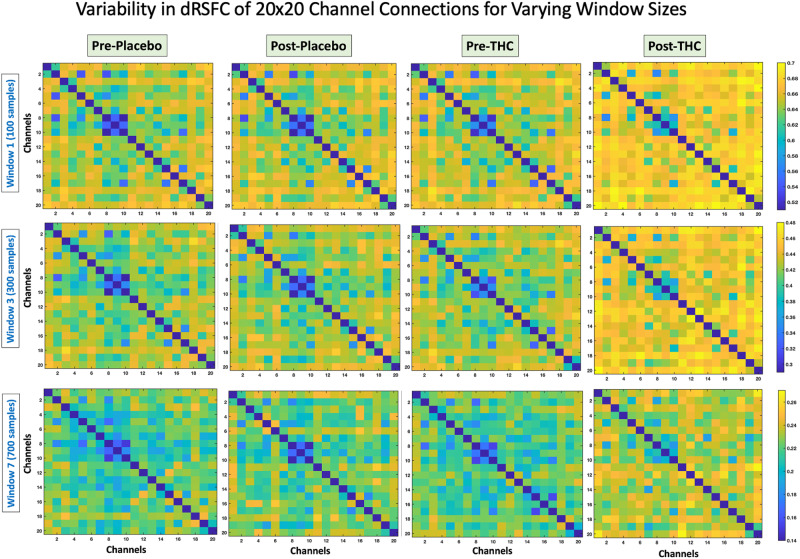
Variability in dynamic RSFC for varying window sizes. Group level variability in dRSFC in 20 × 20 channel pairs for each of the four conditions, pre- and post- placebo and THC. Three representative window sizes are shown, Top: Window 1 (100 samples), Middle: Window 3 (300 samples), Bottom: Window 7 (700 samples). The color bar indicates the mean magnitude of variability (Pearson’s r) across all subjects.

There was widespread increase in dRSFC variability post-THC that was not present during placebo or pre-THC conditions. Variability in dRSFC was measured as the standard deviation of the dRSFC series, thus this variability measure (quantified as Pearson’s r correlation) represents the stability of functional connectivity across short serial windows of time. Figure 3A shows the variability in dRSFC in six channel pairs (Channels 1–12, 6–10, 8–10, 8–14, 8–15, 15–19) post-THC at FDR-*p* < 0.05 (uncorr-p threshold=0.0015) (see Supplementary Table 2 for ANOVA results). As depicted in Fig. 3B, three of these connections (channels 8–10,8–14,8–15) were within the dorsomedial PFC region, while the remaining channels were longer-range connections (channels 15–19, 1–2, and 6–10). As the dRSFC derived using sliding window correlation may differ based on the window size, the variability in these channels were computed with other window sizes, namely, 100, 200, 400, 500, 600, and 700 samples, and increased dRSFC variability following THC was consistent irrespective of window size (see Fig. 4).

### Amplitude of intrinsic low-frequency signal (spectral power)

**Fig. 5:**
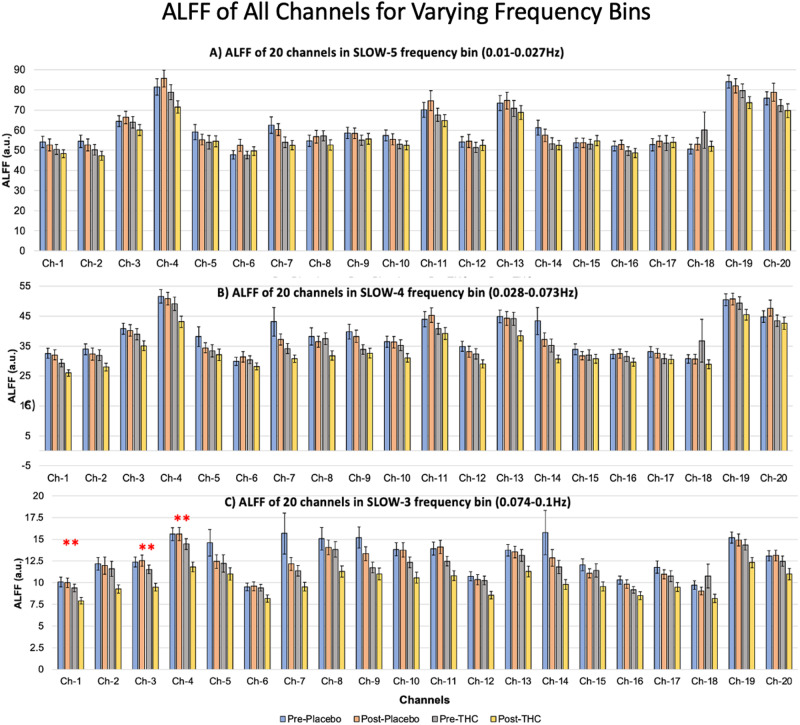
Average spectral power after THC intoxication. ALFF of all channels in (**A**) SLOW-5, (**B**) SLOW-4 and (**C**) SLOW-3 frequency bins for the four conditions. **significant at FDR-*p* < 0.05.

ALFF measures in the SLOW-3 frequency (between 0.073 and 0.1 Hz) was reduced in 16 of 20 channels after THC, based on group level mean values (Fig. 5C). Channels 1 (*p* = 0.039, *η*_*p*_^*2*^ = 0.032, *95% CI*_*ηp2*_: [0,0.11]), 3 (*p* = 0.003, *η*_*p*_^*2*^ = 0.064, *95% CI*_*ηp2*_: [0.007,0.159]), and 4 (*p* = 0.003, *η*_*p*_^*2*^ = 0.067, *95% CI*_*ηp2*_: [0.008,0.163]) were reduced at FDR-p < 0.05 (uncorr-p = 0.0396). ALFF measures did not reveal any significant differences in the SLOW-5 and SLOW-4 frequency bins after FDR correction (Fig. 5A, B).

### Adverse events

Twenty-nine participants reported adverse events that were considered related to THC. The most common were anxiety (13 participants), vomiting (11 participants), nausea (10 participants), and dizziness (10 participants). Dronabinol-induced tachycardia (Heart Rate>100) was observed in fourty-one participants and elevated blood pressure (systolic blood pressure (SBP) > 140) in three participants. These adverse events were considered mild to moderate and were transient. Transient, asymptomatic severe hypertension (SBP > 180) was observed in two participants, correlating with peak drug effect.

## Discussion

This study aimed to determine whether fNIRS could be used to detect effects of acute THC intoxication on the resting-state brain of regular cannabis users. This fNIRS study replicates previous fMRI findings, where we show that RSFC within the PFC, specifically the dorsomedial and dorsolateral PFC, is reduced during acute THC intoxication [26, 28, 29, 48–50]. Additionally, we extend this literature by demonstrating that (1) acute THC increases dRSFC variability in the PFC, (2) the average spectral power of PFC regions in the 0.073–0.1 Hz is reduced following THC, and (3) several of these effects are more pronounced in those with more severe intoxication. Together these findings of reduced RSFC, reduced stability of dRSFC, and reduced power provide a more comprehensive picture of the impact of THC intoxication on prefrontal cortical function, supporting that THC intoxication disrupts PFC connectivity. This picture is consistent with inefficiency in the PFC as a network hub communicating with other brain regions during intoxication. These PFC effects may underlie the cognitive effects of acute THC intoxication. The ability to measure these features of THC intoxication with fNIRS provides a potential avenue for a brain-based, portable, roadside method for detection of impairment from THC.

### Effects of acute THC intoxication on static RSFC

Weakened RSFC correlations and anticorrelations within medial and dorsal PFC regions were observed in participants after oral THC but not placebo. These findings are consistent with a series of studies that predominantly reported hypoconnectivity within brain circuits as a primary response during cannabis intoxication [4, 28]. THC is known to bind to cannabinoid (CB1) receptors in the PFC (among other areas) and alter the GABAergic and glutamatergic mediated processes [27]. Further, subjective ratings of THC intoxication were significantly associated with the magnitude of the connectivity between medial and ventrolateral PFC (Channels 9–16). These findings using fNIRS support prior reports using fMRI that acute cannabis intoxication produces a pattern of decreased functional connectivity between the subcortical and the dorsal attention network (comprising of DLPFC, frontal eye field, and temporal-parietal areas) and between the limbic and cerebellar networks that are strongly associated with the feeling of subjective high [31]; perhaps, suggesting a reduction of top-down attention control and motor coordination during cannabis intoxication [26]. The reduction in correlation (positive connection) within the anterior DMN (left and right medial PFC, channels 7–14) and reduction in anti-correlation (negative connection) between the anterior DMN and non-DMN regions (medial and ventrolateral PFC, channels 9–16) with increasing subjective intoxication further indicates impaired DMN activity at rest after THC, consistent with a previous report that THC administration prevented deactivation of the DMN during cognitive tasks [31]. Further, the high levels of RSFC correlations between channels 7 and 14 after THC in the participants that report least ‘high’, could indicate that the PFC is engaging compensatory control mechanisms in these participants, perhaps in an effort to reduce the psychoactive effects of THC [51]. This is supported by a recent EEG study, which demonstrated that THC was associated with reduced alpha power, suggesting increased cognitive activity or effort, in the frontal lobe [52], which could also indicate the engagement of compensatory neural mechanisms.

### Effects of acute THC Intoxication on dynamic resting-state functional connectivity

The brain is naturally dynamic in its spatiotemporal organization. Here we assess dynamic connectivity across regions of the PFC, which has been associated with vigilance [53], arousal [54], and emotional state [32]. Our findings indicate that THC intoxication increases variability, indicating reduced stability, in the connection strength within PFC regions over time. This suggests that the intoxicating effects of THC may emerge from processes that are not fully captured using traditional static RSFC. Prior whole brain fMRI studies of dRSFC report that people with higher dRSFC variability between FPN and DMN have more cognitive rigidity [55], suggesting that similar processes may be at play with the increased variability following THC observed in this study. The reduced stability in dynamic connectivity may disrupt executive function by reducing the ability of cortical networks to efficiently adapt or reconfigure to salient stimuli [56], impacting higher-order cognitive processes.

### Effects of acute THC intoxication on spectral power, measured by ALFF

THC intoxication was associated with a notable decrease in the ALFF measures of the PFC, particularly in the SLOW-3 frequency. Changes in average spectral power across the lower frequency spectrum indicate changes in the amplitude of spontaneous neuronal oscillations and systemic physiological activity. The three frequency bands in our study (SLOW-5, SLOW-4, and SLOW-3) encompass frequencies below 0.1 Hz, and neuronal oscillations in this frequency range are reported to be primarily associated with gray matter oscillations [57]. A decrease in the spectral power of 0.074–0.1 Hz bin following THC may indicate reduced neural activity or a form of neural suppression or inhibition. While this could reflect decreased excitability or strength of functional connectivity within PFC and between other brain regions, because decreased ALFF measures were observed in most channels, the origin of these effects may be physiological or vascular. Known autonomic effects of acute THC, namely, increased heart rate and systolic blood pressure and decreased heart rate variability [58, 59] were observed in this trial. Interestingly, oscillations at the ~0.1 Hz range are typically composed of Mayer waves, waves of vasomotor oscillations in arterial blood pressure that are influenced by both sympathetic nerve and cardiac activity [60]. Thus, decreased amplitude of 0.074–0.1 Hz frequency could be associated to a shift in power of signals from low frequency to higher frequencies following acute autonomic changes post-THC. These results are consistent with a previous report in which THC increased resting-state signal fluctuations in the right insula, substantia nigra, and cerebellum, which the authors posit was most likely caused by increased spontaneous activity or by increased amplitude of low frequencies [51].

### Implications of an objective marker of acute THC intoxication

These fNIRS-ascertained findings of reduced RSFC and stability of dRSFC in the prefrontal cortical connectome and reduced power in the prefrontal cortex may be hallmarks of acute THC intoxication. A distinction between THC exposure and THC intoxication is important. With THC intoxication, unlike with alcohol, there is a highly variable relationship between blood, breath, or saliva THC or THC metabolite concentration and THC-induced intoxication or impairment that is dependent on the individual level of tolerance to THC [61, 62]. Therefore, it is likely that objective behavior- or brain-based metrics (e.g. eye tracking, cognitive testing [63], and or fNIRS), rather than a per se blood or oral fluid THC or THC metabolite concentration, is required to distinguish impairment due to THC intoxication from exposure without impairment [9]. Our results suggests that certain features of the RSFC of the prefrontal cortex using fNIRS may be useful in developing objective, reliable, and fair tests of impairment from THC, which saliva or blood tests cannot provide.

### Limitations

This study has limitations. First, we focus specifically on prefrontal brain regions, as fNIRS cannot probe deeper structures of the brain that are likely also be affected by THC impairment. The findings of reduced RSFC, reduced stability of dRSFC and reduced power in PFC suggest future study is warranted to investigate whether similar inefficiency in connectivity is present in other brain regions during acute THC intoxication. Second, we did not collect blood that would have allowed for an objective comparison of THC exposure pre-and post-study drug, in addition to dose, because it has been widely shown that blood THC concentrations do not correlate with subjective intoxication [64], and we previously reported that dronabinol dose was not correlated with THC intoxication or impairmen [21, 23]. Third, the current cohort of individuals reported regular cannabis use prior to the study days and therefore may have an adapted acute response to oral THC when compared to drug naïve individuals, such that additional work in people with less regular or more occasional cannabis exposure is needed to understand how these brain measures may adapt or change during processes underpinning tolerance. Finally, we used individualized dosing procedures with the goal of delivering THC doses that were both well tolerated and produced intoxication in each participant. Factors that determine the dose required to produce intoxication (with minimal chance for adverse effects), such as potency of THC used in the prior month and the related degree of tolerance, are challenging to specify in the current regulatory environment, and as factors related to individual differences in THC metabolism are poorly characterized.

## Conclusions

In summary, we used portable fNIRS to demonstrate that acute THC intoxication causes significant changes in brain activity within the prefrontal cortex that include (a) reduced correlations and anticorrelations at rest that correlated with severity of intoxication, indicating reduced top-down attention control and engagement of compensatory mechanisms, (b) more variability in dRSFC over time, that may contribute to a disruption of executive function by reducing the ability of cortical networks to efficiently adapt or reconfigure to salient stimuli, and (c) reduced spectral power, indicating THC disrupts the brain’s normal function in this area, as decreased power is generally associated with neural suppression or inhibition. These neurobiological correlates of THC intoxication severity were measurable using fNIRS and could potentially be incorporated into objective roadside impairment testing. Future study is warranted to investigate how these brain effects of acute THC intoxication relate to cognitive performance and operational impairment.

### Supplementary information

Supplementary material

## Data Availability

All data, code, and materials used in the analyses can be provided by Jodi Gilman and Massachusetts General Hospital pending scientific review and a completed data use agreement/material transfer agreement. Requests for all materials should be submitted to Jodi Gilman.
