# Supplementary material for: Intoxication due to Δ9-tetrahydrocannabinol is characterized by disrupted prefrontal cortex activity
Source: Neuropsychopharmacology. 2024 May 7;49(9):1481–90. doi: 10.1038/s41386-024-01876-5 (PMC11251178; doi:10.1038/s41386-024-01876-5)
Supplement: Supplementary file 1 — Supplementary material [file 41386_2024_1876_MOESM1_ESM.pdf]

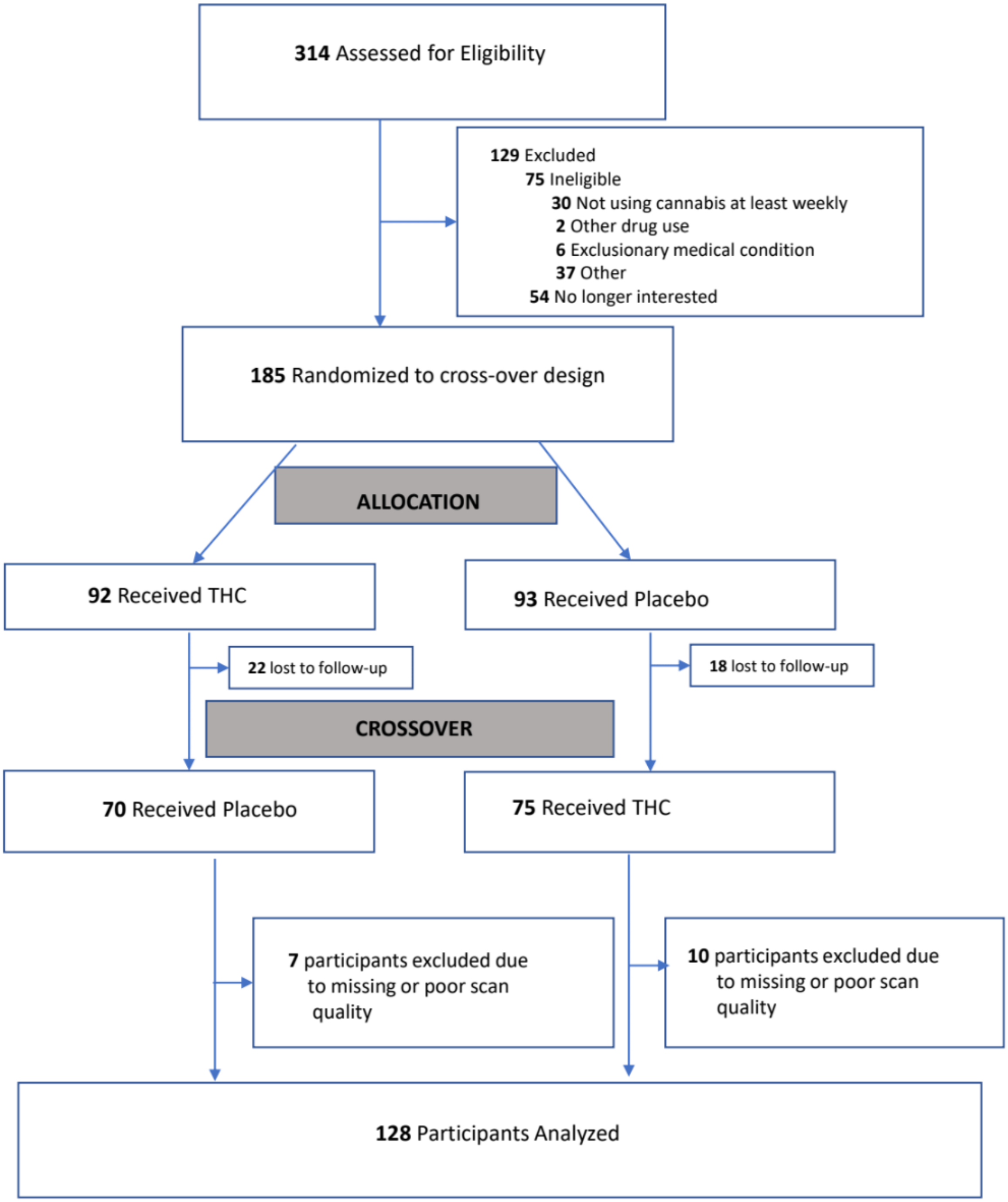


Supplementary Figure 1: CONSORT Flow Diagram

**Table 1. Characteristics of study participants (N = 128)**

| Characteristic | Mean (SD) |
|---|---|
| **Age** | 25.4 (6.4) |
| **Sex** | |
| *Female* | 48.4% (62.0) |
| **Race** | |
| *American Indian or Alaska Native* | 0.8% (1.0) |
| *Asian* | 7.3% (9.0) |
| *Black or African American* | 8.1% (10.0) |
| *Native Hawaiian or Other Pacific Islander* | 0.0% (0.0) |
| *White* | 74.8% (92.0) |
| *More than one race* | 8.9% (11.0) |
| **Ethnicity** | |
| *Hispanic* | 22.4% (28.0) |
| *Non-Hispanic* | 77.6% (97.0) |
| **Years of Education** | 15.3 (2.1) |
| **Age of First Regular Use** | 19.3 (4.1) |
| **Cannabis Use Frequency** | |
| *One or two days per week* | 7.0% (9.0) |
| *Three to five days per week* | 36.7% (47.0) |
| *Six days per week or more* | 56.2% (72.0) |
| **Cannabis Use Occasions Per Day** | |
| *Multiple times per day* | 63.1% (77.0) |
| *No more than once per day* | 36.9% (45.0) |
| **Urine THC-COOH Concentration (ng/mL)** | 238.5 (441.6) |

**Table 2. Channel Connections with Significant Effects in dRSFC Variability**

| | Channel Connection | p-value | Partial eta-squared ($\eta_p^2$) | 95% CI of partial eta-squared |
|---|---|---|---|---|
| 1 | 1-12 | 0.0006 | 0.089 | [0.017,0.191] |
| 2 | 6-10 | 0.0012 | 0.080 | [0.013,0.180] |
| 3 | 8-10 | 0.0006 | 0.088 | [0.016,0.190] |
| 4 | 8-14 | 0.0001 | 0.108 | [0.027,0.215] |
| 5 | 8-15 | 0.0003 | 0.098 | [0.021,0.203] |
| 6 | 15-19 | 0.0015 | 0.077 | [0.012,0.177] |

*Summary of repeated measures ANOVA comparing dRSFC variability pre- and post- placebo and THC at FDR-p<0.05. Uncorrected-p value, partial eta-squared and its 95% CI are provided for channels that survived FDR-based multiple correction at alpha of 0.05.*

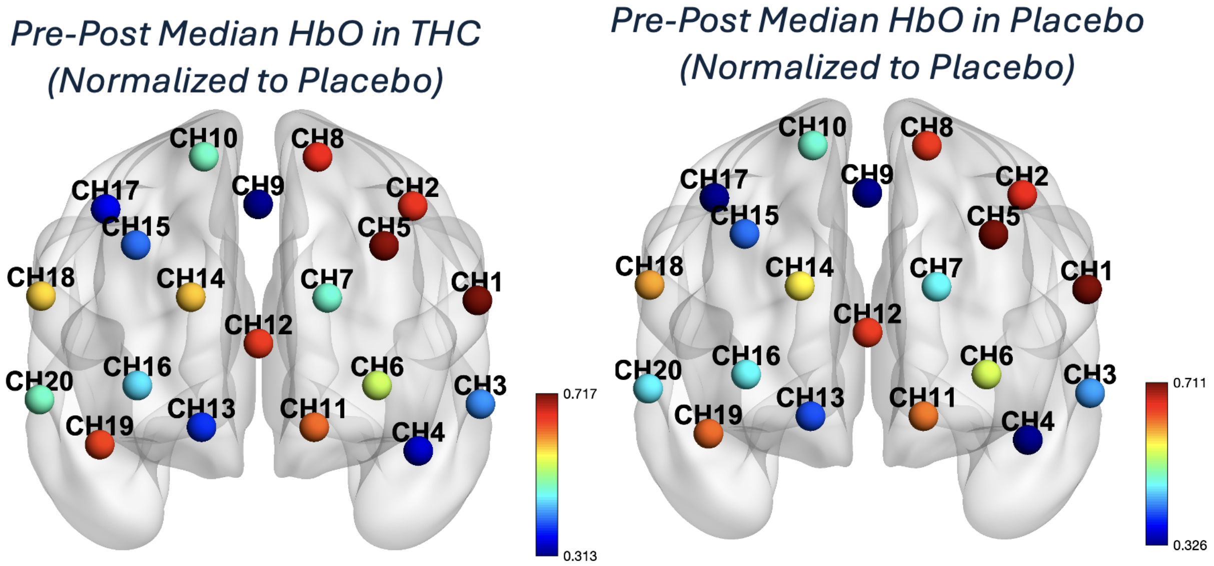


**Supplementary Figure 2: Brain map of the median ΔHbO concentration before vs. after the drug (in %) normalized to the placebo data.** No significant differences were observed between the placebo and THC conditions globally (all channels combined) nor at the individual channel level. We performed this by a) computing the median ΔHbO concentration over the 6-minute duration, b) calculating Δ(pre-post) for placebo and drug, c) normalizing placebo data to 0 to 100%, and (d) normalizing THC data to placebo data. As shown, the median ΔHbO concentration did not differ between post-placebo and post-THC states. The color bar represents the % of median group-level ΔHbO concentration across the various channels.
